# Effect of Mechanical Loads and Surface Roughness on Wear of Silorane and Methacrylate-Based Posterior Composites

**Published:** 2016-11

**Authors:** Masomeh Hasani Tabatabaei, Sakineh Arami, Farnaz Farahat

**Affiliations:** 1 Associate Professor, Dental Research Center, Dentistry Research Institute, Tehran University of Medical Sciences, Tehran, Iran; Department of Operative Dentistry, School of Dentistry, Tehran University of Medical Sciences, Tehran, Iran; 2 Assistant Professor, Department of Operative Dentistry, School of Dentistry, Tehran University of Medical Sciences, Tehran, Iran; 3 Assistant Professor, Department of Operative Dentistry, School of Dentistry, Shahid Sadoughi University of Medical Sciences, Yazd, Iran

**Keywords:** Dental Restoration Wear, Surface Properties, Silorane Composite Resin

## Abstract

**Objectives:**

Dental composite wear in posterior restorations is a concern and is affected by different factors. This study was conducted to evaluate the effect of polishing and mechanical loads on wear of silorane-based and methyl methacrylate-based composites resins.

**Materials and Methods:**

Of each dental composite (Filtek P90 and Filtek P60), 40 samples were fabricated in a polyethylene mold (4mm diameter, 10mm height). According to the finishing and/or polishing protocols (180-grit or 2500-grit silicon carbide papers), the samples of each composite were divided into two groups. Surface roughness (R _ә _) was measured and recorded using a contact profilometer. The weight of each sample was also measured in grams. The wear test was performed in a pin-on-disc device under two different loads (70N, 150N). Afterwards, samples were subjected to profilometry and their weight was measured again. Data were analyzed using t-test and univariate ANOVA. P <0.05 was considered statistically significant.

**Results:**

Higher mechanical load resulted in greater weight loss (P<0.001). Samples polished with 2500-grit papers showed significantly lower Ra changes compared to those polished with 180-grit papers (P<0.001). Filtek P90 had greater weight loss than Filtek P60 except in one condition (180-grit, 70N).

**Conclusions:**

Results showed that wear of posterior composite restorations depends on mechanical load, type of composite resin and surface properties.

## INTRODUCTION

Today, patients’ demand for esthetics as well as their concern about potential toxic effects of mercury have resulted in an increase in use of direct composite restorations instead of amalgam for posterior teeth [1]. Improved biomechanical characteristics of composite resins and conservative preparation of tooth structure are among the most important advantages of composite resins in dental treatments [2].

Wear is a common concern when composite resins are used in posterior teeth [3]. Wear has been mainly reported to be a result of occlusion, chewing, tooth brushing and parafunctional habits (e.g. grinding and clenching) [4]. Clinical signs of composite wear include loss of contour, increased surface roughness, accumulation of plaque and stain, microscopic changes of surface morphology, fatigue and cracks [5]. It has been demonstrated that composite wear is affected by three main factors namely (I) structure of material (related to the type, geometrical properties, size and volume of filler particles), (II) interaction conditions (e.g. force, stress and time), and (III) environmental factors and surface texture (chemical condition, surface topography and temperature) [3, 4].

Recent advances in dental composite resins have focused on polymerization shrinkage and subsequently, polymerization stress. To decrease polymerization shrinkage, resin compounds with high molecular weight have been developed, which have ring-opening monomer systems. Low shrinkage and polymerization stress as well as high stability in aqueous environment are among favorable properties of silorane composites [6].

There are just a few studies investigating the wear of silorane-based composites [7–9]. On the other hand, available reports suggest that the restoration position in the mouth, surface topography and also the magnitude of occlusal forces on some composite restorations produce a significant amount of wear [10–12]. Therefore, this study aimed to assess the effect of mechanical load and surface roughness on the amount of wear of two types of posterior composites namely silorane-based and methyl methacrylate-based composites. The null hypothesis was that wear would not be affected by the magnitude of mechanical load, surface polishing or type of composite resin.

## MATERIALS AND METHODS

### Sample preparation:

Two commercially available composites used in this study were a silorane-based composite (Filtek P90; 3M ESPE, St. Paul, MN, USA) and a methacrylate-based composite (Filtek P60; 3M ESPE, St. Paul, MN, USA). Specifications of these materials are detailed in Table 1.

**Table 1:** Materials used in this study according to the information provided by the manufacturers

** Material **	** Shade **	** Type of filler **	** Mean particle size (μm) **	** Filler (vol%) **	** Resin matrix **	** Manufacturer **	** Batch number **
** Filtek P90 **	A3	Epoxy functional silane-treated SiO2 and ytterbium fluoride	0.47	55	Silorane (oxirane and siloxane)	3M ESPE	195406
** Filtek P60 **	A3	Zirconia/silica (non-silanized)	0.01 to 3.5 average 0.6	61.7	Bis-GMA ^†^ , UDMA ^‡^ , Bis-EMA ^§^	3M ESPE	216614

† Bisphenol A-glycerolate dimethacrylate

‡ Urethane dimethacrylate

§ Bisphenol A-polyethylene glycol diether dimethacrylate

Forty samples were fabricated of each composite material. Fabrication of the samples was performed in a polyethylene mold with 4mm diameter and 10mm height. A thin Mylar strip (Kerr Hawe Neosdent, Bioggio, Switzerland) and the mold were placed on a glass slab with the dimensions of 1×76×76mm.

Afterwards, unpolymerized composite resins were incrementally (2mm layers) packed in the mold, followed by placement of another Mylar strip and glass slab on it. Then, to remove excess material and to achieve a flat surface, gentle finger pressure was applied on the glass slab.

Each increment was cured using a LED light curing unit (Woodpecker Medical Instrument Co., Guilin, China) with a light intensity of 950 mW/cm ^2^ from both sides of the mold for 40 seconds. The samples were removed from the mold and cured again from all four directions for 20 seconds. Twenty samples from each composite group were finished under water irrigation using 180-grit silicon carbide waterproof abrasive papers (Matador, Remscheid, Germany). The surface of the other samples was polished under water irrigation by consequent use of 180, 250, 400, 800, 1000, 2000 and 2500-grit abrasive papers. To reduce the probability of micro-crack formation, each abrasive paper was used for 10 seconds [13]. In order to simulate clinical conditions and to reduce the potential risks of dust aerosols, the polishing procedures were performed under water spray. In addition, possible harmful effects of finishing and polishing on restoration surface were minimized in wet environment.

### Experiments:

After finishing and polishing, all samples were cleaned in an ultrasonic bath (Starsonic 35; Liarre, Bologna, Italy). The surface roughness of each sample was measured by a contact profilometer (T-8000 Hommelwerk; Jentopik, Jena, Germany). The average surface roughness is the amount of prominences and depressions measured on the composite surface in a cross-section [14]. A diamond tip at a speed of 0.5 mm/second and force of 4N scanned the surface. The average amount of surface roughness (Ra) was recorded in micrometers.

Before performing wear tests, the weight of each sample was measured in grams by a digital scale (Mettler Toledo, Mississauga, Canada) with 0.0001g precision. The wear test was performed using a pin-on-disc device in artificial saliva (Bioxtra, Cambrige, Canada). While composite samples were fixed in the pin section of the wear device, aluminum oxide discs were substituted in the disc section rotating by a motor (rotating speed: 0.2 m/s). The motion radius and slipping distance was 22 mm and 500 m, respectively.

During the wear process, half of the polished or unpolished samples of each composite were subjected to mechanical load of 70N and the other half of the samples were subjected to mechanical load of 150N. Therefore, the wear test was done in eight groups with 10 samples per each group.

After performing the wear tests, the samples were weighed and weight loss was calculated. In addition, Ra values were measured by the profilometer and the difference with the baseline condition was calculated.

### Statistical analysis:

Data were analyzed using SPSS version 18 (SPSS Inc., IL, USA). For investigating the effect of three factors namely type of material, mechanical loads and polishing on weight loss, initially two and three-way ANOVA were used. But because of significant interactions (P=0.404), t-test was applied. To evaluate the effect of the three factors on Ra changes, three-way ANOVA was used. P<0.05 was considered statistically significant.

## RESULTS

The average amounts of weight loss of samples during wear test in different groups were calculated (Table 2). The results showed that in both composites and in both polishing grits, higher mechanical load resulted in greater weight loss (P<0.001, Table 3). Comparing weight loss between different polishing grits for the studied materials and loads showed that only the difference between groups 6 and 8 was significant (P=0.032, Table 4).

**Table 2:** Mean and standard deviation of weight loss after wear test (n=10)

** Group **	** Material **	** Polishing (grit) **	** Load (N) **	** Weight loss (g) **
** 1 **	Filtek P90	180	70	0.0037 ± 0.0018 ^ a ^
** 2 **	Filtek P90	180	150	0.0111 ± 0.0025 ^ b ^
** 3 **	Filtek P90	2500	70	0.0032 ± 0.0008 ^ a ^
** 4 **	Filtek P90	2500	150	0.0107 ± 0.0021 ^ b ^
** 5 **	Filtek P60	180	70	0.0027 ± 0.0010 ^ af ^
** 6 **	Filtek P60	180	150	0.0087 ± 0.0018 ^ c ^
** 7 **	Filtek P60	2500	70	0.0020 ± 0.0006 ^ df ^
** 8 **	Filtek P60	2500	150	0.0068 ± 0.0015 ^ e ^

Identical letters indicate no significant difference (P>0.05)

**Table 3:** Comparison of weight loss between the two applied loads

** Material **	** Polishing (grit) **	** Load (N) **	** Group **	Weight loss (g) (Mean ± SD^*^)	** P-value **
** Filtek P60 **	180	70	5	0.0027 ± 0.0010	<0.001
		150	6	0.0087 ± 0.0018	
	2500	70	7	0.0020 ± 0.0006	<0.001
		150	8	0.0068 ± 0.0015	
** Filtek P90 **	180	70	1	0.0037 ± 0.0018	<0.001
		150	2	0.0111 ± 0.0025	
	2500	70	3	0.0032 ± 0.0008	<0.001
		150	4	0.0107 ± 0.0021	

* Standard deviation

**Table 4:** Comparison of weight loss between the two polishing grits

** Material **	** Load (N) **	** Polishing (grit) **	** Group **	Weight loss (g) (Mean ± SD^*^ )	** P-value **
** Filtek P60 **	70	180	5	0.0027 ± 0.0010	0.13
		2500	7	0.0020 ± 0.0006	
	150	180	6	0.0087 ± 0.0018	0.032
		2500	8	0.0068 ± 0.0015	
** Filtek P90 **	70	180	1	0.0037 ± 0.0018	0.45
		2500	3	0.0032 ± 0.0008	
	150	180	2	0.0111 ± 0.0025	0.68
		2500	4	0.0107 ± 0.0021	

* Standard deviation

When weight loss of Filtek P60 and Filtek P90 in defined loads and polishing grits was compared, the results showed that only the difference between groups 1 and 5 was not significant (P=0.159, Table 5).

**Table 5:** Comparison of weight loss between the two materials

** Load (N) **	** Polishing (grit) **	** Material **	** Group **	Weight loss (g) (Mean ± SD^*^)	** P value **
70	180	Filtek P60	5	0.0027 ± 0.0010	0.159
		Filtek P90	1	0.0037 ± 0.0018	
	2500	Filtek P60	7	0.0020 ± 0.0006	0.003
		Filtek P90	3	0.0032 ± 0.0008	
150	180	Filtek P60	6	0.0087 ± 0.0018	0.026
		Filtek P90	2	0.0111 ± 0.0025	
	2500	Filtek P60	8	0.0068 ± 0.0015	<0.001
		Filtek P90	4	0.0107 ± 0.0021	

* Standard deviation

**Fig. 1:**
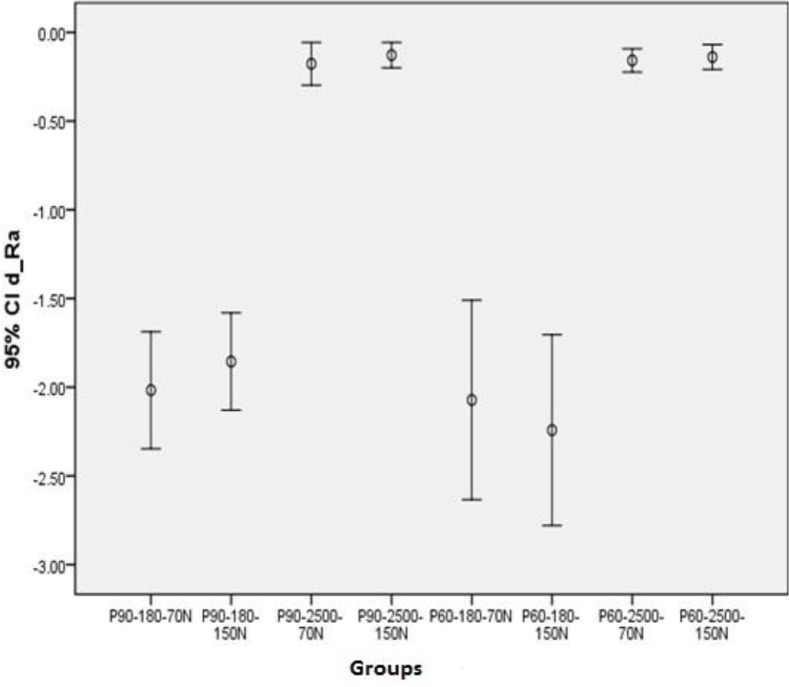
Mean Ra change in different groups

The difference in Ra values in different groups was calculated and statistically analyzed before and after the wear test. The mean changes in Ra are shown in Figure 1. Ra changes were the greatest in group 6 and the lowest in group 4.

Samples polished with 2500-grit paper showed significantly lower Ra changes compared to those polished with 180-grit paper (P<0.001). No significant association was observed between Ra changes and studied materials (P=0.28) or mechanical loads (P= 0.88).

## DISCUSSION

In spite of the beliefs regarding wear resistance of composite restorations, the available limited studies show that wear resistance is a great concern in extensive restorations with direct occlusal contacts and in patients with bruxism and clenching [15]. According to the amount of wear, restorations may fail due to reasons such as submargination and surface roughness changes [16]. This study was done in two-body wear conditions that only simulated one of the clinical wear conditions. There are different methods for evaluating the quality and quantity of composite wear [16–18]. In the present study, we used weight loss and profilometer. Artificial saliva was also used in order to simulate the clinical conditions. In addition, it has been reported that saliva provides ideal lubrication and reduces wear [4]. In this study, 70N and 150N loads were used, which are within the range of masticatory loads [14]. The results of the present study showed that by increasing the mechanical load, the weight loss due to wear increased significantly. Lambrechts et al, [19] showed that the enamel wear due to 78N load was two times greater than that due to the application of 20N load. Ghazal and Kern [20] also revealed that as the load increased, composite and enamel wears increased, too. Heintze et al, [21] evaluated surface deterioration of dental materials after simulated tooth brushing in relation to brushing force and revealed that abrasion of dental composites is affected by the magnitude of the applied load during simulated tooth brushing. Shabanian and Richards [11] also revealed that in a wear machine, higher loads (0 to 9.95kg) resulted in greater wear of Z100 composite, conventional glass ionomer cement and resin-modified glass ionomer cement as well as enamel. The friction between the restorative material and its antagonist is effective on the wear behavior. The friction coefficient is affected by vertical force (occlusal load) and geometric parameters such as the texture of the abrasive surface and contact area. Greater forces result in higher friction coefficients and therefore, the wear increases [22]. On the other hand, under greater loads, the bond between the composite filler and matrix becomes more sensitive to degradation and therefore, by increasing the loads, the wear increases as well [10]. The results of this study showed that regardless of the material type and mechanical load, the roughness (R _ә _) changes in the samples, which were polished with 2500-grit paper were significantly less. The effects of polishing of the two materials and mechanical loads are different due to the interaction effect of polishing with the material type and mechanical load. Samples, which were polished with 2500-grit paper showed lower weight loss, but it was only significant in Filtek P60 composite under 150N load. Turssi et al, [12] showed that the abrasive wear of mini-filled and nanofilled composites was not related to surface roughness. They suggested that the surface irregularities had a little effect on three-body abrasion. However, they mentioned that the duration of their experiments might be the probable reason for masking the effect of polishing on wear. Ratanapridakul et al, [23] reported that polished materials show more wear than unpolished materials. They concluded that rotary polishing equipment creates scratches on the material surface and thus, decreases abrasion resistance. In the present study, the lower Ra changes in 2500-grit polishing compared to 180-grit might be related to the friction between the composite and antagonist. A polished surface is smoother; thus, it has lower surface roughness and the friction between composite and antagonist reduces. This reduction leads to lower composite wear [24]. Analysis of the data showed that weight loss of Filtek P90 was significantly more than that of Filtek P60 in our study. The Ra changes in the two materials were close to each other; thus, the material type was not significantly effective on Ra changes.

There is no agreement on the wear resistance of packable composites. Some believe that these kinds of composites have high wear resistance [25, 26] while some are of the opinion that the wear of these composites is more than that of non-packable resins. Wang et al, [27] showed that weight loss due to wear in Filtek P60 was more than that of Alert (a packable composite), Surefil and Z100 and less than that of Silux Plus (micro-filled), Prodigy Condensable (packable) and Solitaire (packable). In addition, Turssi et al, [28] reported that wear of Filtek P60 was more than that of Z250 and Charisma (hybrid) and less than that of Tetric Ceram HB (packable). Another study revealed that posterior composite resins, designed for stress-bearing areas (Filtek P60 and Filtek P90), did not display higher wear resistance than the universal dental composites. They also showed that the wear of composites including the new matrix monomers and conventional matrix composites with similar filler volume was not significantly different and thus the abrasive wear of Filtek P90 was more than that of Filtek P60 [7]. Hahnel et al, [8] showed that wear behavior of silorane-based composite and ormocer was similar. The disagreement between studies is probably because of different conditions and types of tests performed.

Matrix type, shape, size, volume and distribution of filler particles, filler hardness, silanization and the degree of conversion (DC) are effective factors on composites wear [4, 29]. A smaller size of filler particles, more silanization and more volume percentage of filler result in less composite wear [30, 31]. Filtek P90 and Filtek P60 have different matrixes; Filtek P90 matrix contains silorane, an innovative monomer system, obtained from the reaction of oxirane and siloxane molecules. The ring-opening mechanism in oxirane results in low polymerization shrinkage, and presence of siloxane molecule increases hydrophobicity [7]. But Filtek P60 composite contains Bis-GMA, Bis-EMA and UDMA. This difference may lead to different wear behaviors [32]. The DC may also be contributory. It has been shown that cationic polymerization reaction in the silorane-based composites does not progress as fast as that in methacrylate-based composites. Thus, composites with different monomers have different DC that finally affects their wear resistance [28]. The size of filler particles in Filtek P60 is bigger than that in Filtek P90, but the volume percentage of the filler in Filtek P60 is higher. The higher filler content results in preservation of the organic matrix and reduction of wear [33]. The diameter of the Filtek P60 filler particles is approximately 0.6μ. These fillers are uniformly distributed as discrete particles. Jorgensen [33] reported that when the distance between adjacent fillers is approximately 0.1μ, the matrix is protected against wear, which may be the reason for superior wear resistance of Filtek P60 in our study.

The reason for the lack of difference in Ra changes between the two materials (in spite of the difference in the amount of weight loss) could be that during contact between the antagonist and material surface, some filler particles are rubbed off while some others are pressed into the surface; as a result, wear and particle aggregation occur at the same time [24]. Thus, although Filtek P90 wears more, there is no clear difference in surface changes with Filtek P60. This study was done in two-body wear conditions that only simulated one of the clinical wear conditions. More in vitro researches and clinical trials should be done in future to better elucidate this topic.

## CONCLUSION

Under tested experimental conditions, mechanical load had a pronounced effect on posterior composite wear. Filtek P90 had greater wear than Filtek P60, and surface topography affected the Ra changes during wear process.
